# Multicentre, double-blind, crossover trial to identify the Optimal Pathway for TreatIng neurOpathic paiN in Diabetes Mellitus (OPTION-DM): study protocol for a randomised controlled trial

**DOI:** 10.1186/s13063-018-2959-y

**Published:** 2018-10-22

**Authors:** Dinesh Selvarajah, Jennifer Petrie, David White, Steven Julious, Oscar Bortolami, Cindy Cooper, Mike Bradburn, Amanda Loban, Helen Bowler, Lizzie Swaby, Katie Sutherland, Solomon Tesfaye, on behalf of the OPTION-DM group, Satyan Rajbhandari, David Bennett, Rajiv Gandhi, Edward Jude, Gerry Rayman, Prashanth Vas, Ravikanth Gouni, Didier Bouhassira, Michelle Horspool, Martin Johnson, Tracey Young

**Affiliations:** 1 Department of Oncology and Human Metabolism, Medical School, University of Sheffield, Sheffield, UK; 2 Clinical Trials Research Unit, University of Sheffield, Sheffield, UK; 3 Medical Statistics Group, School of Health and Related Research, University of Sheffield, Sheffield, UK; 4 Sheffield Teaching Hospitals NHS Foundation Trust, Sheffield, UK

**Keywords:** Diabetes, Painful diabetic neuropathy, Pregabalin, Duloxetine, Amitriptyline, Crossover trial

## Abstract

**Background:**

The number of people with diabetes is growing rapidly. Diabetes can cause nerve damage leading to severe pain in the feet, legs and hands, which is known as diabetic peripheral neuropathic pain (DPNP). In the UK, the National Institute for Health and Care Excellence (NICE) recommends amitriptyline, duloxetine, pregabalin or gabapentin as initial treatment for DPNP. If this is not effective, adding one of the other drugs in combination with the first is recommended. NICE points out that these recommendations are not based on robust evidence. The OPTION-DM randomised controlled trial has been designed to address this evidence deficit, with the aims of determining the most clinically beneficial, cost-effective and tolerated treatment pathway for patients with DPNP.

**Methods/design:**

A multicentre, double-blind, centre-stratified, multi-period crossover study with equal allocation to sequences (1:1:1:1:1:1) of treatment pathways. Three hundred and ninety-two participants will be recruited from secondary care DPNP centres in the UK. There are three treatment pathways: amitriptyline supplemented with pregabalin, pregabalin supplemented with amitriptyline and duloxetine supplemented with pregabalin. All participants will receive all three pathways and randomisation will determine the order in which they are received. The primary outcome is the difference between 7-day average 24-h pain scores on an 11-point NRS scale measured during the final follow-up week of the treatment pathway. Secondary outcomes for efficacy, cost-effectiveness, safety, patient-perceived tolerability and subgroup analysis will be measured at week 6 and week 16 of each pathway.

**Discussion:**

The study includes direct comparisons of the mainstay treatment for DPNP. This novel study is designed to examine treatment pathways and capture clinically relevant outcomes which will make the results generalisable to current clinical practice. The study will also provide information on health economic outcomes and will include a subgroup study to provide information on whether patient phenotypes predict response to treatment.

**Trial registration:**

ISRCTN17545443. Registered on 12 September 2016.

**Electronic supplementary material:**

The online version of this article (10.1186/s13063-018-2959-y) contains supplementary material, which is available to authorized users.

## Background

In August 2015, Diabetes UK announced that the prevalence of diabetes had increased by 60% over the previous decade to 3.3 million. Diabetic peripheral neuropathic pain (DPNP) is a serious complication affecting up to 20–26% of these patients [1, 2]. With the prevalence of diabetes set to increase by epidemic proportions over the next decade, DPNP will pose a major treatment challenge [3, 4]. With advanced disease the pain can extend above the feet and may involve the whole of the legs, and when this is the case there is often upper limb involvement also. Moderate-to-severe unremitting lower limb pain is present in over 70% of sufferers [2, 5] and causes insomnia, poor Quality of Life (QoL), unemployment and depression [6–9].

The mainstay of treatment for DPNP is pharmacotherapy. Recent National Institute for Health and Care Excellence (NICE) guidance (173) [10] recommends a choice of amitriptyline, duloxetine, pregabalin or gabapentin as initial treatment. All are licensed treatments for DPNP except amitriptyline, which has been used off-license for more than 25 years. There is moderate evidence for the efficacy of each drug based on Cochrane reviews [11–14] and meta-analyses [15–17], but the best we can hope for any monotherapy is 50% pain relief in 50% of patients [10]. This is often accompanied by side effects (dry mouth, constipation, sedation, dizziness, falls, nausea, oedema, etc.) in around 10–20% depending on dose. NICE recommends combination treatment if initial treatment is not effective (the majority) [10]. However, as NICE points out recommendations are not based on robust evidence as: (1) there are few well-designed head-to-head studies comparing the first-line drugs and their combinations; (2) most studies were flawed with inadequate power, inappropriate endpoints, short duration of follow-up and (3) many randomised controlled trials (RCTs) lacked appropriate health-related QoL (HRQL) measures including functionality and failed to measure impact of drug-related adverse effects on health economics and QoL [10]. An RCT is needed to address these deficiencies.

The aims of the OPTION-DM study will be to determine the most clinically beneficial, cost-effective and tolerated treatment pathway for patients with DPNP. The study has been designed to have direct clinical applicability in the management of DPNP following completion.

### Study objectives

1. To evaluate if at least one of the three pathways is superior to the other pathways in terms of pain symptoms, quality of life and cost-effectiveness
2. To evaluate if at least one monotherapy is superior to a different monotherapy in improving pain symptoms
3. To describe Adverse Event and Serious Adverse Event data for the different treatment pathways for DPNP
4. To conduct an exploratory analysis to investigate whether there are patient phenotypes that predict response to treatment

## Methods/design

### Study design

OPTION-DM is a multicentre, double-blind, centre-stratified, multi-period crossover study with equal allocation to sequences (1:1:1:1:1:1) of treatment pathways. Three hundred and ninety-two participants will be recruited from secondary care DPNP centres in the UK. A list of participating centres can be found at the end of this paper. Recruitment is expected to take place over 12 months beginning in October 2017. Follow-up will continue for another 12 months.

The study contains an internal pilot with stop-go criteria to assess its feasibility. The recruitment and retention will be reviewed in relation to targets agreed with the funder after 6 months of recruitment. If met, an assessment of attrition to the trial is scheduled after 12 months of recruitment.

The study protocol was written in accordance with the Standard Protocol Items: Recommendations for Interventional Trials (SPIRIT) guidelines (see Additional file 1: SPIRIT Checklist).

### Participants

A number of approaches will be used to identify potential participants: hospital database searches will be completed at each of the study centres; potential patients may be identified during routine hospital appointments at a study centre; the general practitioner (GP) patient registers at around 80 GP surgeries aligned to the study centres will be checked for patients with a diagnosis of diabetes and prescriptions for neuropathic pain medications; Participant Identification Centres (PIC) will be utilised; community podiatry services will be engaged to encourage referrals of potential patients, if applicable; details of the study will be advertised through the use of posters and leaflets in various clinics (for example, diabetes outpatient clinics or GP surgeries); the study will be advertised in a number of locations, such as on charity websites, in local libraries, local newspapers and via local radio stations to inform potential participants about the study.

Potentially eligible participants will be provided with the participant information sheet. Informed consent will be obtained by a medically qualified site investigator trained in Good Clinical Practice.

### Eligibility criteria

#### Inclusion criteria

1. Participant aged ≥ 18 years
2. Neuropathic pain affecting both feet and / or hands for at least 3 months or taking pain medication for neuropathic pain for at least 3 months
3. Bilateral distal symmetrical neuropathic pain confirmed by the Douleur Neuropathique 4 (DN4) questionnaire at the screening visit [18]
4. Bilateral distal symmetrical polyneuropathy confirmed by a modified Toronto Clinical Neuropathy Score (mTCNS) > 5 at the screening visit [19]
5. Stable glycaemic control (HbA1c < 108 mmol/mol)
6. Participants will have a mean total pain intensity of at least 4 on an 11-point numeric rating scale (NRS; with 0 being ‘no pain’ and 10 ‘worst pain imaginable’) during 1 week off pain medications (baseline period)
7. Willing and able to comply with all the study requirements and be available for the duration of the study. This will be a 1-year study in which all participants will undergo all treatment pathways regardless of treatment response and this point will be made clear
8. Willing to discontinue current neuropathic-pain-relieving medications
9. Informed consent form for study participation signed by participant

#### Exclusion criteria

1. Non-diabetic symmetrical polyneuropathies
2. History of alcohol/substance abuse which would, in the opinion of the investigator, impair their ability to take part in the study
3. History of severe psychiatric illnesses which would, in the opinion of the investigator, impair their ability to take part in the study
4. History of epilepsy
5. Contraindications to study medications
6. Pregnancy/breast feeding or planning pregnancy during the course of the study
7. Use of prohibited concomitant treatment that could not be discontinued
8. Use of high-dose morphine equivalent (> 100 mg/day)
9. Liver disease (AST/ALT > 2 times upper limit of normal)
10. Significant renal impairment (estimated glomerular filtration rate (eGFR) < 30 ml/min/1.73m^2^)
11. Heart failure New York Heart Association (NYHA) ≥ class II
12. Clinically significant cardiac arrhythmias on 12-lead ECG or current history of arrhythmia
13. Patients with a recent myocardial infarction (< 6 months prior to randomisation)
14. Postural hypotension (reduction of > 20 mmHg)
15. Prostatic hypertrophy or urinary retention to an extent which would, in the opinion of the investigator, be a contraindication to the study medication
16. Patients with other painful medical conditions where the intensity of the pain is significantly more severe than their diabetic peripheral neuropathic pain (patients will not be excluded if the pain is transient in nature)
17. Any suicide risk as judged by the investigator or as defined by a score of ≥ 2 on the suicide risk questionnaire
18. Significant language barriers which are likely to affect the participant's understanding of the medication schedule or ability to complete outcome questionnaires
19. Concurrent participation in another clinical trial of an investigational medicinal product
20. Major amputations of the lower limbs
21. Active diabetic foot ulcers

### Washout and baseline period

After providing consent, participants will be instructed on how to washout neuropathic pain medication. The dose will be tapered for 3 days with complete washout for 1–4 days at the investigator's discretion. If the participant is on combination therapy then all drugs will be tapered at once. For participants taking 50-100-mg morphine equivalent the dose will be tapered over a period of up to 2 weeks.

Following the initial washout period, participants will enter the baseline period for 1 week. No neuropathic pain medication is permitted during this week with the exception of paracetamol. From the daily pain scores collected during the baseline period, a mean for the week will be determined and used in subsequent analysis.

### Interventions

#### Treatment pathways

The OPTION-DM will study three treatment pathways:

- Amitriptyline supplemented with pregabalin (A-P Pathway)
- Duloxetine supplemented with pregabalin (D-P Pathway)
- Pregabalin supplemented with amitriptyline (P-A Pathway)

#### Randomisation

Participants will be centrally randomised in the study by the study team at site using the Clinical Trials Research Unit (CTRU) online randomisation system. All participants will receive all three treatment pathways (Table 1). Randomisation will determine the order in which they receive the treatment pathways. Participants will be assigned to one of the six sequences based on a predetermined randomisation schedule stratified by study site using permuted blocks.

**Table 1:** Treatment sequences (A = amitriptyline, P = pregabalin, D = duloxetine)

	Treatment pathway 1	Treatment pathway 2	Treatment pathway 3
Sequence 1	A-P	D-P	P-A
Sequence 2	A-P	P-A	D-P
Sequence 3	D-P	A-P	P-A
Sequence 4	D-P	P-A	A-P
Sequence 5	P-A	A-P	D-P
Sequence 6	P-A	D-P	A-P

#### Treatment phases

**Fig. 1:**
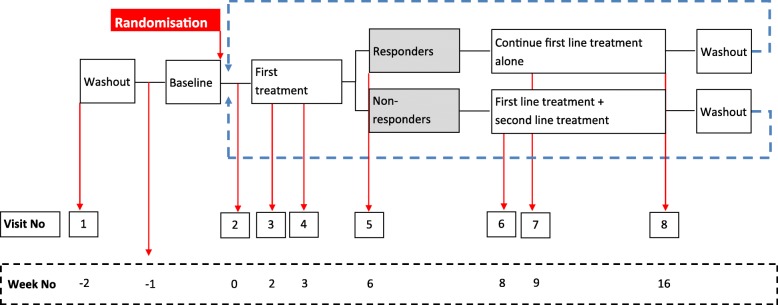
Participant flow

Each treatment pathway has two treatment phases (Fig. 1). During the first treatment phase, participants will receive monotherapy with the first-line treatment in the pathway. This will last for a total of 6 weeks, including the dose-titration phase.

At the week-6 follow-up visit a decision will be taken to either continue on monotherapy or to add second-line treatment as combination therapy based on the 7-day average 24-h pain NRS score during the week preceding the study visit. Participants will be divided into ‘responders’ (pain score ≤ 3) and ‘non-responders’ (pain score > 3) and this will be used to guide treatment during the second treatment phase.

The second treatment phase will last for a total of 10 weeks. Non-responders will commence combination therapy with the addition of second-line treatment for 10 weeks, including the dose-titration phase. Responders will continue on monotherapy for the remainder of treatment phase 2, but this decision may be reversed up to week 13 in the event that a participant becomes a ‘non-responder’ later in the second treatment phase. The dose titration will follow the same schedule.

At the week-16 follow-up visit, participants will be advised to taper-down study medication (3 days) and stop the medication completely (4 days) before commencing the next treatment pathway. The taper dose will be one dose level below the maximum tolerated dose. Participants on dose level 1 will not require a taper dose and will stop study medication completely for 7 days. The first and second treatment phases will be repeated until the participant completes all three pathways.

#### Dose titration

**Fig. 2:**
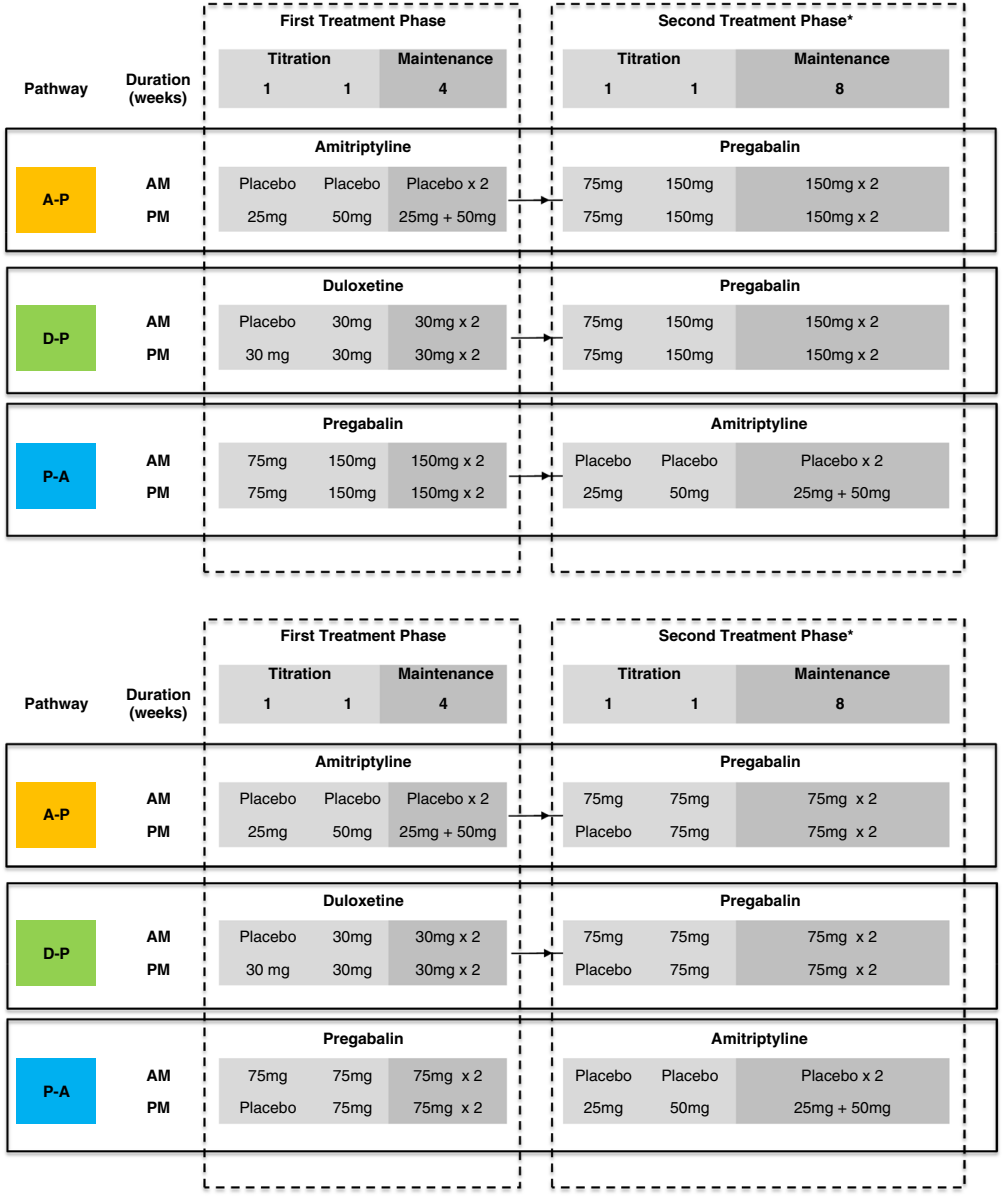
**a** Dosing and titration schedule for each treatment pathway (*standard pregabalin dosing, estimated glomerular filtration rate (eGFR) ≥ 60 ml/min*). *Participants continue on the maintenance dose of drug from the first treatment phase for the duration of the second treatment phase. **b** Dosing and titration schedule for each treatment pathway (*reduced pregabalin dosing, eGFR 30–59 ml/min*). *Participants continue on the maintenance dose of drug from the first treatment phase for the duration of the second treatment phase

There will be three dose levels for each drug and participants will always start on the lowest dose level of each drug. The schedule of dose escalation will be identical in each treatment pathway, see Fig. 2a and b. Patients with renal insufficiency will receive a modified dosing schedule: eGFR will be measured at screening and at week 16 of each pathway and patients whose eGFR was 30–59 ml/min at their most recent test will receive a lower dose of pregabalin. Pharmacy will be informed of the latest eGFR result with each prescription in order to ensure that the correct dose of pregabalin is dispensed.

During the first 2 weeks of each treatment phase, the dose will be escalated towards the maximum tolerated dose or maximum permitted dose, whichever is first, based on treatment response (based on the 24-h pain NRS score) and side effect profile.

During the weekly telephone calls and scheduled study visits, the research nurse will evaluate response to treatment and adverse effects to guide dose titration accordingly. If patients are receiving adequate pain relief (24-h pain NRS score ≤ 3) at dose level 1 or 2 then the dose will not be increased further. Patients will also be asked to rate any reported side effects. These will be graded (mild, moderate or severe) and whether side effects are tolerable or intolerable. Any severe or intolerable side effects will require a medication review (i.e. consider dose reduction or discontinuation).

#### Switching treatment during a pathway

At the week-6 visit if there was no change in pain scores from the pre-treatment pathway (baseline), participants will switch to the second-line treatment in the treatment pathway.

If there is significant intolerance to first-line treatment; for example, due to side effects which are severe or which the patient describes as intolerable, participants can switch to the second-line treatment in the treatment pathway as a monotherapy. In this situation the switch can be made immediately, at any time, without the need to washout the first-line treatment. The second-line treatment will be continued as a monotherapy for the remainder of the treatment pathway; i.e. up to the week-16 visit. If there is significant intolerance to the second-line treatment in the pathway, the participant will stop the study treatment but will remain in the study for follow-up.

### Blinding

OPTION-DM is a double-blind study and blinding of medication will be maintained with over-encapsulated drugs and matching placebos. The treating physician will be aware of the dose level but not the treatment itself. Due to the complex dosing schedule, the pharmacist at each study centre will be unblinded and a member of staff at Sheffield CTRU responsible for site monitoring will also be unblinded.

Emergency unblinding can be performed where the knowledge of the treatment allocation would change the participant’s clinical management or to facilitate safety reporting to the regulatory authority and Research Ethics Committee.

### Adherence

Participants will be provided with detailed guidance regarding how to take their study medication. This will be reinforced with written instructions and participants will be directed to complete a daily medication diary to record which doses they have taken.

Participants will be asked to return all bottles of study medication, including empty bottles and any unused medication. These will be reviewed and the remaining capsules counted to monitor adherence to study treatment. The study nurse will provide further guidance to participants if there is concern about adherence levels.

### Concomitant medications

Participants will maintain their current schedule of treatment throughout the duration of the study. Changes to concomitant medications will be documented at each study visit. Participants may take paracetamol 1 g (up to a maximum dose of four times a day (QDS)) for pain throughout the study period.

The following concomitant medications are prohibited during the study period: opioid analgesia, capsaicin cream/high-dose capsaicin patches, lidocaine patches, anti-inflammatory medications (e.g. diclofenac, colecoxib), other antiepileptic medications (e.g. carbamazepine), other antidepressant medications (e.g. SSRIs, MAOIs), other neuropathic pain medications (e.g. venlafaxine, intravenously administered (IV) lignocaine, etc.), use of any medications that could lead to potentially serious interactions with study medications.

### Blood sample collection

Blood samples will be stored for future research which may include genetic analysis. Samples will be obtained at the same time as other study blood samples from participants who have given additional (optional) consent. The blood will be frozen and stored locally before being shipped to a central laboratory.

### Study procedures

**Fig. 3:**
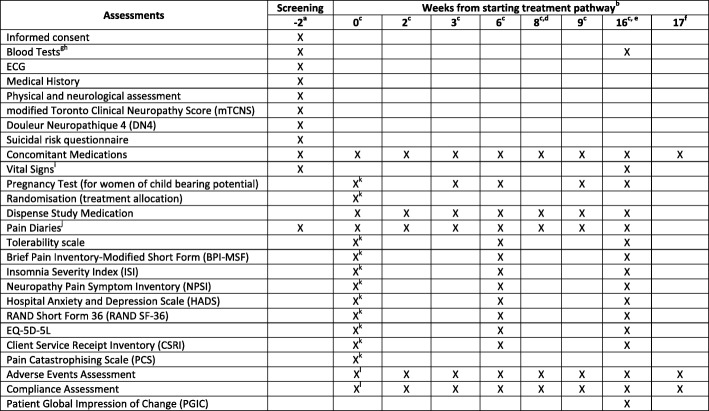
Study assessment schedule (Standard Protocol Items: Recommendations for Interventional Trials (SPIRIT) Figure). ^a^This visit is only required prior to randomisation, i.e. before starting the first treatment pathway. ^b^ Between scheduled study visits, the research nurse will contact the participant by telephone each week (a minimum of once per week). The nurse will confirm compliance with medication and remind the participant to complete study diaries/questionnaires. ^c^ Visits must normally be within ± 2 days of the scheduled visit date. Scheduled visit dates relate to the date of the previous visit. Where this is impossible, e.g. due to bank holidays or patient availability. ^d^ Week-8 visit only required for participants on combination treatment. ^e^ At the week-16 visit, participants will be given instructions to tape-off the current study treatment. Visits from week 0 to week 16 will be repeated until all 3 pathways have been completed. ^f^ Week 17 is only applicable following the final pathway. ^g^ FBC, urea and electrolytes, liver function tests, glycosylated haemoglobin A1c and serum creatinine. ^h^ hole blood sample to be collected and stored for future research. The sample can be obtained at the same time as any scheduled blood test for the study. Please refer to the *OPTION-DM Sample Collection Manual* for details. ^i^ Height (at week − 2 only), weight, heart rate and blood pressure (lying and standing). ^j^ To be completed by participants daily during the study, starting during the washout period. Pain scores may also be collected via daily text messages where participants have given additional consent for this. ^k^ Only required at week 0 of pathway 1, i.e. randomisation visit. ^l.^Not required at week 0 of pathway 1, i.e. randomisation visit

The study assessment schedule (SPIRIT Figure; Fig. 3) below details the assessments required during the course of one treatment pathway. All participants will complete three treatment pathways and this schedule will be repeated from week 0 to week 16 until all three pathways are complete. Week 17 will only be relevant at the end of the final pathway.

### Primary outcome

The primary outcome is the difference between 7-day average 24-h pain (evaluated at patient level) on an 11-point NRS scale (0 = no pain and 10 = worst pain imaginable) measured during the final follow-up week of the treatment cycle (week 16) among pathways. The NRS 24-h average pain is now considered the ‘gold standard’ for the assessment of neuropathic pain and has been employed in almost all well-designed neuropathic pain studies over the past 10 years [15, 20, 21].

### Secondary outcomes

#### Efficacy

1. Difference between 7-day average 24-h pain (evaluated at patient level) on an 11-point NRS scale at week 6 among monotherapies
2. Difference between RAND short form 36 (RAND SF-36) physical mean scores (evaluated at patient level) at week 16 among pathways [22]
3. Difference between RAND SF-36 physical mean scores (evaluated at patient level) at week 6 among pathways [22]
4. Difference between RAND SF-36 mental mean scores (evaluated at patient level) at week 16 among pathways [22]
5. Difference between RAND SF-36 mental mean scores (evaluated at patient level) at week 6 among pathways [22]
6. Difference between Hospital Anxiety and Depression Scale (HADS) mean anxiety scores (evaluated at patient level) at week 6 among pathways [23].
7. Difference between Hospital Anxiety and Depression Scale (HADS) mean anxiety scores (evaluated at patient level) at week 16 among pathways [23]
8. Difference between Hospital Anxiety and Depression Scale (HADS) mean depression scores (evaluated at patient level) at week 6 among pathways [23]
9. Difference between Hospital Anxiety and Depression Scale (HADS) mean depression scores (evaluated at patient level) at week 16 among pathways [23]
10. Difference in proportion of patients having treatment success (30%) at week 16 among pathways. Treatment success is defined as a reduction in 30% value of 7-day average NRS score at follow-up compared to baseline
11. Difference in proportion of patients having treatment success (50%) at week 16 among pathways. Treatment success is defined as a reduction in 50% value of 7-day average NRS score at follow-up compared to baseline

Difference in Brief Pain Inventory – modified short form (BPI-MSF) measure of pain interference with function total score (evaluated at patient level) at week 6 among pathways [24]

12. Difference in BPI-MSF measure of pain interference with function total score (evaluated at patient level) at week 16 among pathways [24]
13. Difference in Insomnia Severity Index (evaluated at patient level) total score at week 6 among pathways [25]
14. Difference in Insomnia Severity Index (evaluated at patient level) total score at week 16 among pathways [25]
15. Difference in Patient Global Impression of Change (evaluated at patient level) at week 16 among pathways [26]
16. Difference in proportion of care pathway preferred by participants at week 50

#### Cost-effectiveness

17. EuroQoL-5D-5 L: the EQ-5D is a routinely used generic HRQL instrument. It is the preferred instrument for assessing HRQL by NICE, and the newer five-level (EQ-5D-5 L) instrument offers increased sensitivity as opposed to the original three-level version [27]
18. A modified version of the Client Service Receipt Inventory (CSRI): the CSRI is a routinely used instrument to capture health resource use and personal expenses. Unnecessary questions will be removed to reduce participant burden [28]

#### Safety

20. Frequency and proportion of patients reporting at least one Adverse Event for each of the pathway. Additionally, the relationship to intervention (Definite, Probable, Possible, Unlikely, Unrelated, Not assessable) will be reported (frequency and proportion)
21. Frequency and proportion of Adverse Events for each of the pathways
22. Listing of Adverse Events for each of the pathways
23. Frequency and proportion of patients reporting at least one Serious Adverse Event for each of the pathways. Additionally, these characteristics will be summarised (frequency and proportion): intensity (Mild, Moderate, Severe), relationship (Definite, Probable, Possible, Unlikely, Unrelated, Not assessable), is SUSAR, is Death
24. Frequencies of Serious Adverse Events for each of the pathways
25. Listing of Serious Adverse Events for each of the pathways

#### Subgroup

Neuropathic Pain Symptom Inventory (NPSI) questionnaire for subgroup analysis relating pain phenotype to treatment response [29]. There is emerging evidence that treatment response may be determined by a patient’s pain phenotype [30–32]. In particular, these outcomes will be evaluated:

26. Difference between ‘Burning (superficial) spontaneous pain’ NPSI mean subscores – (evaluated at patient level) at week 6 among pathways
27. Difference between ‘Burning (superficial) spontaneous pain’ NPSI mean subscores – (evaluated at patient level) at week 16 among pathways
28. Difference between ‘Pressing (deep) spontaneous pain’ NPSI mean subscores – (evaluated at patient level) at week 6 among pathways
29. Difference between ‘Pressing (deep) spontaneous pain’ NPSI mean subscores – (evaluated at patient level) at week 16 among pathways
30. Difference between ‘Paroxysmal pain’ NPSI mean subscores – (evaluated at patient level) at week 6 among pathways
31. Difference between ‘Paroxysmal pain’ NPSI mean subscores – (evaluated at patient level) at week 16 among pathways
32. Difference between ‘Evoked pain’ NPSI mean subscores – (evaluated at patient level) at week 6 among pathways
33. Difference between ‘Evoked pain’ NPSI mean subscores – (evaluated at patient level) at week 16 among pathways
34. Difference between ‘Paresthesia/dysaesthesia’ NPSI mean subscores – (evaluated at patient level) at week 6 among pathways
35. Difference between ‘Paresthesia/dysaesthesia’ NPSI mean subscores – (evaluated at patient level) at week 16 among pathways
36. Difference between NPSI mean total scores – (evaluated at patient level) at week 6 among pathways
37. Difference between NPSI mean total scores – (evaluated at patient level) at week 16 among pathways

### Patient-perceived tolerability

38. Difference between tolerability (evaluated at patient level) on an 11-point NRS scale at week 16 among pathways-.
39. Difference between tolerability (evaluated at patient level) on an 11-point NRS scale at week 6 among monotherapies

### Sample size

A 1-point change in an individual on the NRS scale is considered a minimum clinically important difference [33]. Hence, the proportion of patients improving by at least 1 point would seem a suitable outcome. However, we have based the sample size calculation on a continuous outcome, the mean change between groups in order to maintain power [34]. We have chosen a mean change at the population level of 0.5 points between groups based on the effect size previously reported for comparison of two active interventions for neuropathic pain in a crossover study [35]. Based on Normal Distribution Theory we estimate that a 0.5-point shift in population means will lead to an additional 8% of individual patients achieving a 1-point improvement [36]. We have also used a conservative, Bonferroni-corrected significance of 1.67% in order to retain an overall 5% false-positive probability for finding a significant pairwise comparison. Using a within-patient SD of 1.65 [35], an alpha of 0.0167 and 90% power, we require 294 evaluable patients [37]. Assuming a 25% dropout rate 392 patients will be randomised to ensure that 294 patients are expected to complete the study.

### Withdrawals

An individual participant may stop treatment early for any of the following reasons:

- Unacceptable toxicity
- Withdrawal of consent for treatment by the participant
- Inter-current illness which prevents further treatment
- Any alteration in the participant’s condition which justifies the discontinuation of treatment in the investigator’s opinion
- Pregnancy

Participants will be followed up as per the trial schedule until the end of the current treatment pathway, provided they are willing. A discussion will also take place to clarify whether the participant is discontinuing all study treatment or whether they wish to return for the next treatment pathway.

When a participant stops treatment in the OPTION-DM study, they will return to their usual care provider for treatment outwith the study. At the end of the trial, when the final analysis has been completed, the CTRU will provide participating sites with the unblinded treatment allocations for each of their participants. The site staff will be responsible for contacting each of the participants to notify them of the treatment allocations. It will then be a clinical decision between the participant and their usual care provider as to which treatment they receive.

### Data collection and management

Participant confidentiality will be respected at all times and the principles of the UK Data Protection Act (DPA) will be followed. The study will use the CTRU’s in-house data management system (Prospect) for the capture and storage of study-specific participant data. Access to Prospect is controlled by usernames and encrypted passwords, and a comprehensive privilege management feature will be used to ensure that users have access to only the minimum amount of data required to complete their tasks. A member of staff at each site will enter data from source documents into the study-specific Prospect database when available. After data have been entered, electronic validation rules are applied to the database on a regular basis; discrepancies are tracked and resolved through the Prospect database. Questionnaires are self-completed by participants and entered onto the study database by the research nurse. Data will be stored and managed in accordance with CTRU Standard Operating Procedures (SOPs).

### Statistical analysis

The statistical analysis will be reported according to Consolidated Standards of Reporting Trials (CONSORT) guidelines [38] and using an intention-to-treat approach as the primary analysis. As three pairwise comparisons will be performed, all statistical tests will be two-tailed at a 1.67% significance level. The primary outcome and other continuous outcomes will be analysed using a random-effects model with participant, treatment, sequence and period entered into the model. Participant will be entered as a random term. Contrasts will be used to evaluate the difference in means. Three 98.33% confidence intervals for the difference on treatment effect will be reported as well as the associated *P* value.

In case of missing data, the missing data mechanism will be explored and multiple imputation may be applied as a sensitivity analysis as appropriate. Other sensitivity analyses will be performed in order to evaluate the robustness of the primary analyses [39].

A logistic regression will be undertaken to analyse binary outcomes using a model similar to that for the continuous outcomes. Differences between treatment groups will be reported as odds ratios with associated 98.33% confidence intervals and *P* values. Full details of the statistical analyses will be specified in a detailed Statistical Analysis Plan.

### Monitoring

Conduct of this study will be governed by three committees. An independent Trial Steering Committee (TSC) will oversee the conduct of the trial. An independent Data Monitoring and Ethics Committee (DMEC) will monitor participant safety. A Trial Management Group (TMG) will be responsible for the day-to-day running of the trial. The roles and responsibilities of the groups are included in the group charter or terms of reference.

CTRU will undertake monitoring visits at each investigator site before, during and after the trial. Central monitoring will also be utilised to review data, consent forms and accountability logs.

Details of Adverse Events will be collected at each study visit or telephone call. Serious Adverse Events will be assessed by the local investigator and reported to Sheffield CTRU within 24 h.

### Ethics and dissemination

Ethical approval for the study was given by Yorkshire and the Humber – Sheffield Research Ethics Committee (reference number: 16/YH/0459). Any protocol amendments will be reviewed and approved by the Ethics Committee and regulatory authority as applicable before being notified to all relevant parties.

The results of the trial will be disseminated in peer-reviewed scientific journals and clinical and academic conferences. A lay summary of the results will be sent directly to participants. The results will also be freely available via the funding body’s journal website [40] and a summary will be published on the Sheffield CTRU website.

### Patient public involvement

The Diabetes PPI Panel at Sheffield Teaching Hospitals reviewed the study at the proposal development stage. They were supportive of the proposal including the study design and they contributed to the choice of endpoints for the study. In particular, they were pleased with the efficient crossover design as participants will receive active treatment during all treatment phases. Although the duration of the study is long, they felt that participants are more likely to remain in the study as active treatment is received. The panel were later involved in the development of the patient information sheet, consent form and study medication diary. We also have a patient representative as a member of the TSC; therefore, we will have ongoing patient involvement in the management of the study.

## Discussion

DPNP is a distressing and disabling condition which is often intractable to treatment. Unfortunately current treatment only achieves meaningful pain relief in two out of three patients. Despite much research there are no current or emerging treatments that alter the natural course of the disease. This study is timely as it addresses an important clinical need by providing evidence as to which is the most clinically beneficial and cost-effective treatment pathway for DPNP.

### Why examine treatment pathways?

The examination of a treatment pathway as a whole is the most efficient and applicable to current UK clinical practice. This is because most patients are started on monotherapy and will require a second agent added in combination within a few months [41]. Only a very small minority will either have sufficient benefit from monotherapy and will not need another agent, or will not tolerate monotherapy (or monotherapy is completely ineffective) and will be switched to another agent. Thus, OPTION-DM, which will examine the whole treatment pathway, will capture more clinically relevant outcomes than artificially designed, head-to-head monotherapy or combination studies. Hence, the outcomes of this study will be readily generalisable to current UK clinical practice.

### Why exclude gabapentin?

There is clear rationale for not studying two α-2-δ agonists (pregabalin and gabapentin) as:

1. The evidence for gabapentin is only derived from one reasonable-quality RCT (4-week titration and 4-week treatment phase) [42] compared to eight RCTs in pregabalin and evidence supported by meta-analysis [15]
2. Gabapentin is a thrice daily drug
3. Gabapentin, unlike pregabalin does not have linear pharmacokinetics and requires a long titration period of up to 2 months [43] to avoid toxicity

### Which treatment pathways?

We will not examine the pathway of pregabalin supplemented by duloxetine because of the COMBO-DN findings [44]. In this study, there was no difference in pain reduction if pregabalin was added to duloxetine or vice versa [44]. However, duloxetine was superior to pregabalin as an initial treatment, is a once daily preparation and is also the cheaper option in the UK. There is thus a good rationale for starting patients on duloxetine and then adding pregabalin in combination. Finally, as both amitriptyline and duloxetine are antidepressants there is little rationale for combining both.

### Efficient design with 16-week treatment pathways

This will be an efficiently designed head-to-head, crossover RCT [21] with each patient undergoing all pathways. The duration of monotherapy in each pathway is at least 6 weeks, an adequate duration to assess treatment effect and whether combination therapy is indicated [21, 43]. The subsequent 10-week combination therapy in patients with partial benefit from monotherapy will be adequate to assess stabilised treatment outcomes [44]. The COMBO-DN study used fixed-dose-titration regimens regardless of treatment response. This resulted in a dropout rate of 17% during monotherapy and 12% during combination therapy [44]. The present trial is a pragmatic RCT employing a flexible dosing regimen to achieve maximum-tolerated doses based on individual responses; we envision that this will reduce the dropout rate. The use of rescue medication, frequent clinic and telephone contacts and the need for active therapy we envision will further reduce dropout rates. Completion rates will be monitored on an ongoing basis.

### Trial status

The current protocol is version 7.0, 1 February 2018. The study began recruiting in November 2017 and is estimated to be completed in October 2018. Follow-up will continue for a further 12 months. We anticipate that the results will be available in early 2020.

## Additional file

Additional file 1: Standard Protocol Items: Recommendations for Interventional Trials (SPIRIT) Checklist. (DOC 119 kb)

## Abbreviations

BPI-MSF: Brief Pain Inventory – modified short form
CSRI: Client Service Receipt Inventory
CTRU: Clinical Trials Research Unit
DN4: Douleur Neuropathique 4
DPNP: Diabetic peripheral neuropathic pain
eGFR: Estimated glomerular filtration rate
HADS: Hospital Anxiety and Depression Scale
ISI: Insomnia Severity Index
mTCNS: Modified Toronto Clinical Neuropathy Score
NICE: National Institute for Health and Care Excellence
NPSI: Neuropathy Pain Symptom Index
NRS: Numeric rating scale
PCS: Pain Catastrophising Scale
PGIC: Patient Global Impression of Change
PIC: Participant Identification Centre
QoL: Quality of life
RAND SF-36: RAND short form 36
RCT: Randomised controlled trial
SOP: Standard operating procedure
